# Enhanced Bilateral Accelerated Theta Burst Stimulation for Comorbid Anxiety and Depressive Disorders: The Seville Protocol

**DOI:** 10.31083/AP45254

**Published:** 2025-12-16

**Authors:** Á. Moleón-Ruiz, P. Álvarez de Toledo, L. García-Fernández, MI. Pérez-Aquino, J. Narbona-Antunez, S. Jiménez-Fernández, L. Gutiérrez-Rojas, M. Martín-Bejarano, R. Rodriguez-Jimenez

**Affiliations:** ^1^Psychiatry Service, Hospital Virgen del Rocío, 41013 Seville, Spain; ^2^Instituto Andaluz de Salud Cerebral, 41010 Seville, Spain; ^3^Clinical Medicine Department, Universidad Miguel Hernández, 03202 Alicante, Spain; ^4^Psychiatry Department, Hospital Universitario de San Juan, 03550 Alicante, Spain; ^5^CIBERSAM-ISCIII (Biomedical Research Networking Centre for Mental Health), 28029 Madrid, Spain; ^6^Department of Psychiatry and Neurosciences Research Group (CTS-549), Institute of Neurosciences, University of Granada, 18071 Granada, Spain; ^7^Psychiatry Service, Hospital San Cecilio, 18014 Granada, Spain; ^8^Complutense University of Madrid (UCM), 28040 Madrid, Spain; ^9^Research Institute of Hospital Universitario 12 de Octubre (imas12), 28041 Madrid, Spain

**Keywords:** treatment resistant depression, anxiety, transcranial magnetic stimulation, theta burst stimulation

## Abstract

**Background:**

Treatment-resistant depression (TRD) with comorbid anxiety affects up to 30% of patients and frequently fails to respond to conventional therapeutic approaches. The Seville Protocol is a novel, accelerated, high-dose, bilateral theta burst stimulation (TBS) paradigm combining intermittent TBS (iTBS) and continuous TBS (cTBS), specifically designed to address both depressive and anxiety symptoms in TRD.

**Methods:**

This retrospective study was conducted at the Andalusian Institute of Brain Health (Seville, Spain). All participants received the Seville Protocol, consisting of neuronavigated iTBS applied to the left and cTBS to the right dorsolateral prefrontal cortex (DLPFC), delivered at high intensity (110–133.5% of the resting motor threshold) over 30 sessions within three weeks. Symptom severity was assessed at baseline and post-treatment using the Hamilton Depression Rating Scale (HAM-D) and the Hamilton Anxiety Rating Scale (HAM-A). Treatment efficacy was analyzed using the Wilcoxon signed-rank test, and logistic regression models were applied to identify predictors of response and remission.

**Results:**

A total of 64 patients diagnosed with TRD and comorbid anxiety were included in the analysis. The Seville Protocol led to significant improvements in both HAM-D and HAM-A scores (*p* < 0.001). Response rates were 45.3% for depression (95% Confidence Interval (CI) = 33.7–57.4) and 48.4% for anxiety (95% CI = 36.6–60.4), while remission rates were 29.7% for depression (95% CI = 19.9–41.8) and 23.4% for anxiety (95% CI = 14.7–35.1). Logistic regression analysis suggested that a positive family history of mental disorders was associated with a lower likelihood of depression response (β = –1.49, 95% CI = –2.98 to –0.18, *p* = 0.033); however, this association did not remain significant after false discovery rate (FDR) correction (adjusted *p* = 0.298).

**Conclusions:**

The Seville Protocol appears to be a feasible, practical, and time-efficient neuromodulation approach for patients with TRD and comorbid anxiety. These findings support the potential utility of accelerated bilateral TBS in this population, although further studies are needed to validate the findings and assess their broader applicability.

## Main Points

∙ The Seville Protocol is an accelerated, high-dose bilateral TBS treatment specifically designed for patients with treatment-resistant depression (TRD) with comorbid anxiety.

∙ In 64 patients, 30 sessions over 3 weeks produced significant improvements, with response rates of ~45% for depression and 48% for anxiety, and remission rates of 30% and 23%, respectively.

∙ The protocol was well tolerated and offers a shorter, scalable alternative to standard repetitive transcranial magnetic stimulation (rTMS) in real-world clinical practice.

## 1. Introduction

Treatment-resistant depression (TRD), defined as an inadequate response to at least two different classes of antidepressant treatments administered at appropriate doses and durations, affects approximately 30–40% of individuals with major depressive disorder (MDD), leading to significant distress, disability, and increased caregiver burden, as well as substantial economic costs for healthcare systems [1, 2]. Compared to individuals with non-resistant depression, patients with TRD experience significantly lower health-related quality of life in both mental and physical domains, greater functional impairment, reduced work productivity, and markedly higher healthcare utilization and costs [3]. Moreover, comorbid anxiety disorders, including panic disorder, generalized anxiety disorder, social anxiety disorder, and obsessive-compulsive disorder, have been identified as predictors of poorer treatment outcomes and slower remission rates in depressive populations [4, 5].

Importantly, TRD with anxious distress is considered a particularly difficult-to-treat subtype, often associated with greater symptom burden and worse prognosis [5]. One therapeutic alternative is repetitive transcranial magnetic stimulation (rTMS), a non-invasive neuromodulation technique that has been approved by both the U.S. Food and Drug Administration (FDA) and CE-marked in Europe as a medical device for the treatment of TRD [6, 7].

Standard rTMS protocols typically involve daily stimulation sessions applied to the left dorsolateral prefrontal cortex (DLPFC) at high frequencies (10–20 Hz) over a 4- to 6-week period, delivering between 3000 and 4000 pulses per session [8]. Although effective, their long duration, requiring daily sessions over several weeks, combined with variable individual responses, has prompted research into more time-efficient alternatives. Among these, theta burst stimulation (TBS) has emerged as a promising option. Continuous TBS (cTBS) applied to the right DLPFC reduces cortical excitability and may alleviate anxiety-related symptoms, whereas intermittent TBS (iTBS) applied to the left DLPFC enhances cortical excitability and has demonstrated antidepressant efficacy [9, 10, 11, 12]. Evidence also suggests that bilateral TBS, combining left iTBS and right cTBS, achieves superior outcomes compared with unilateral protocols or conventional high-frequency rTMS [13].

In parallel, recent advancements in electric field modeling have underscored the importance of optimizing stimulation parameters to maximize therapeutic efficacy. Traditional dosing approaches, generally based on 80%–120% of the resting motor threshold (RMT), have been revisited, revealing that significantly higher intensities are often necessary to achieve equivalent stimulation in prefrontal areas due to anatomical variability [14, 15]. These findings highlight the need for individualized dosing to reduce the risk of suboptimal stimulation and enhance clinical outcomes.

To date, few studies have evaluated the clinical outcomes of accelerated, high-dose, high-intensity bilateral TBS in TRD populations, particularly those with comorbid anxiety symptoms. Pioneering protocols by Chen *et al*. [16] and Stöhrmann *et al*. [17] have demonstrated the feasibility of bilateral TBS approaches, employing 600 pulses per target at 120% RMT, with two to three sessions daily over short treatment periods, yielding promising response and remission rates.

The primary aim of the present study was to evaluate the clinical response to the Seville Protocol in patients with TRD and comorbid anxiety symptoms. This novel intensive neuromodulation protocol, developed by our group, builds on previous bilateral TBS paradigms but innovates by delivering higher stimulation intensities (110%–133.5% RMT) [14, 15]. and greater pulse doses per session [18], aiming to achieve a more rapid and robust clinical response. Moreover, recognizing the clinical relevance of comorbid anxiety in TRD and its impact on outcomes, the protocol was specifically tailored to address both depression and anxiety symptoms concurrently. While other protocols such as Stanford SAINT (Stanford Accelerated Intelligent Neuromodulation Therapy) [19] have more frequent delivery schedules (ten sessions five consecutive days), such intensity may pose logistical and tolerability challenges in many clinical settings. The Seville Protocol was therefore developed to strike a balance between total stimulation dose and clinical feasibility, delivering 3600 pulses per session, three days per week, over a three-week period. As a secondary objective and given prior studies reporting their influence on rTMS outcomes [20, 21], we examined the relationship between sociodemographic and clinical baseline factors on treatment response.

## 2. Methods

### 2.1 Subjects

This study involved a retrospective chart review of patients diagnosed with TRD and comorbid anxiety symptoms treated at the Andalusian Institute of Brain Health in Seville, Spain. Diagnoses were established according to DSM-5-TR criteria by board-certified psychiatrists. Inclusion criteria were: (1) age between 18 and 80 years; (2) a diagnosis of TRD, defined as failure to respond to at least two adequate antidepressant trials; and (3) the presence of clinically moderate-to-severe anxiety symptoms. Exclusion criteria included a history of neurological disorders, active substance abuse (except nicotine or caffeine) or any contraindication to transcranial magnetic stimulation (TMS) [22]. This naturalistic approach was adopted to maximize external validity to routine clinical settings. Consequently, all eligible patients who initiated the Seville Protocol during the study period and met the inclusion criteria were included.

The study was conducted in accordance with the Declaration of Helsinki and approved by the Ethics Committee of Research Institute of Hospital Universitario 12 de Octubre (imas12) (Internal Code: 25/255). All data were anonymized prior to analysis to ensure confidentiality, and participants had provided informed consent for the use of their clinical information for research purposes.

### 2.2 The Seville Protocol

Stimulation was delivered using the MagPro X100 system with MagOption (MagVenture, Farum, Denmark), equipped with a Cool D-B80 coil (MagVenture, Farum, Denmark). We administered accelerated cTBS and iTBS over the bilateral DLPFC. Sessions were guided using neuronavigation based on each participant’s T1-weighted structural magnetic resonance imaging (MRI), co-registered to standard montreal neurological institute (MNI) space, with the Visor2^TM^ software (ANT Neuro, Enschede, Netherlands). Targets in the DLPFC were defined a priori using MNI coordinates (x, y, z): right DLPFC [35, 39, 31] and left DLPFC [–39, 34, 37]. Specifically, accelerated cTBS was directed to the right DLPFC, and iTBS to the left DLPFC, each delivering 1800 pulses per target, for a total of 3600 pulses per session. Participants received three to four sessions per day, three consecutive days per week, over a three-week period, resulting in a total of 30 sessions. Asymmetrical stimulation intensities were used, 110% of RMT for cTBS and 133.5% RMT for iTBS. The selection of these intensities was informed by both prior research and electric field modelling studies. Right cTBS at 110% RMT aligns with established protocols for reducing excitability in this region in anxiety and depression [3, 23]. The use of 133.5% RMT for left iTBS was based on modelling data showing that such intensity is necessary to achieve electric fields in the prefrontal cortex equivalent to those generated by 100% RMT over the motor cortex [14, 15].

**Fig. 1.**
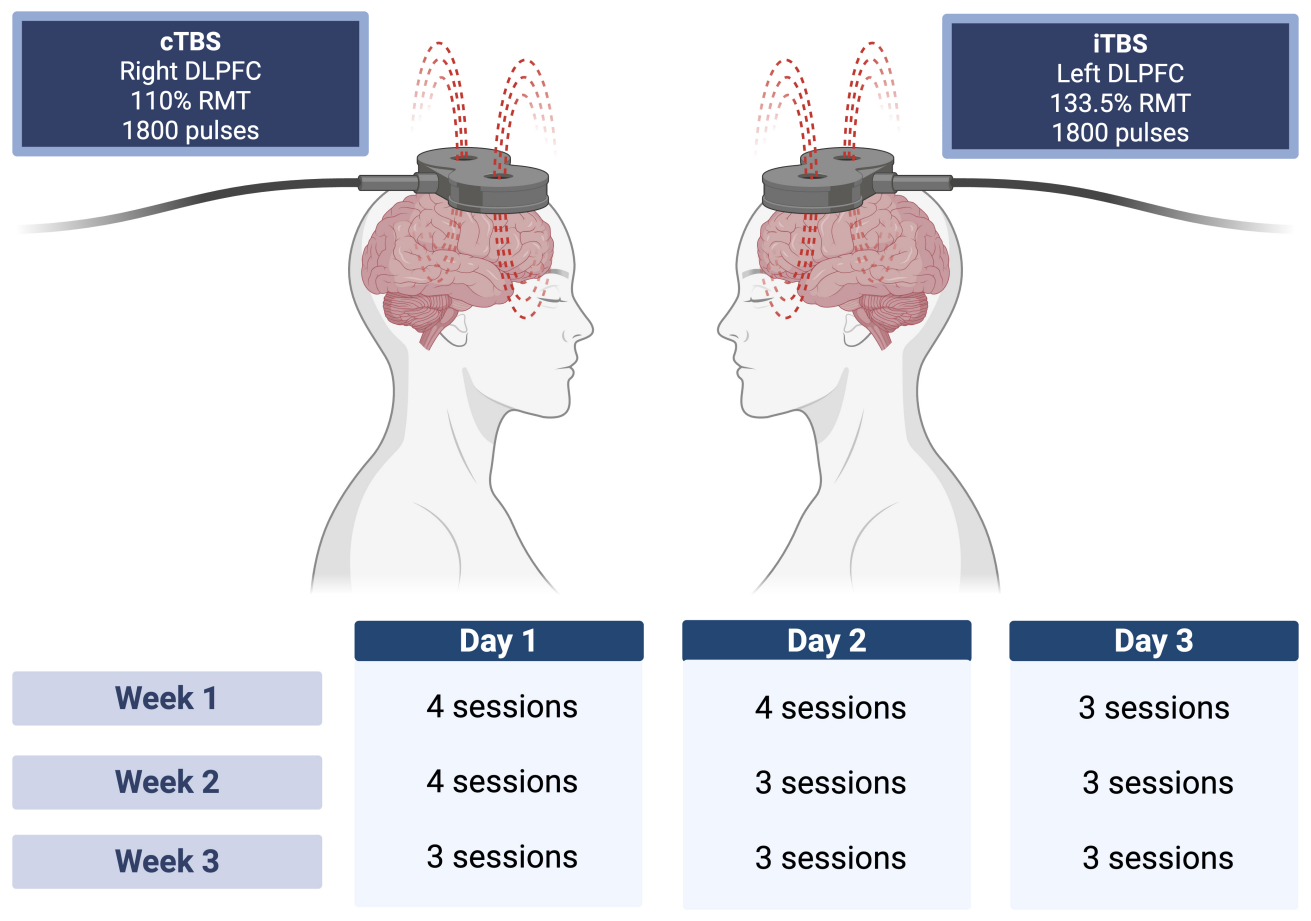
**Visual summary of the Seville Protocol**. Bilateral stimulation is applied with cTBS to the right DLPFC and iTBS to the left DLPFC (1800 pulses per target). The lower panel illustrates the distribution of sessions across the three-week treatment schedule. cTBS, continuous high-dose bilateral; DLPFC, dorsolateral prefrontal cortex; iTBS, intermittent TBS; RMT, resting motor threshold. Created in BioRender (https://www.biorender.com/).

Fig. 1 provides a visual summary of the stimulation targets and session schedule.

To ensure treatment stability across the TBS protocol, no changes were implemented in the concomitant pharmacological regimens of patients, and no new psychotherapeutic interventions were initiated during the study period.

### 2.3 Instruments

Depressive symptoms were assessed using the 17-item Hamilton Depression Rating Scale (HAM-D) [24] and the 14-item Hamilton Anxiety Rating Scale (HAM-A) [25], respectively. Both are clinician-administered instruments, widely used to evaluate the severity of psychiatric symptoms. Assessments were conducted at baseline and after completing the TMS treatment to measure clinical change by a board-certified psychologist with clinical expertise.

Total scores range from 0 to 52 for the HAM-D and from 0 to 56 for the HAM-A, with higher scores indicating greater symptom severity. In both scales, scores below 17 are typically interpreted as mild severity, 18–24 as mild to moderate, and 25–30 as moderate to severe symptomatology.

In this study, patients were included if they presented with clinically moderate-to-severe anxiety symptoms, as judged by their treating psychiatrist, based on the HAM-A score and the presence of clinically evaluated functional impairment. While a formal cutoff of ≥18 was generally applied, individuals scoring slightly below this threshold (e.g., 13–17) were also considered eligible if anxiety symptoms resulted in significant functional impairment, consistent with clinical judgment and the real-world heterogeneity of TRD with anxious features. No formal cut-off was applied for depressive symptoms on the HAM-D.

### 2.4 Statistical Analysis

Descriptive statistics were computed for all sociodemographic and clinical variables. Continuous variables are reported as means and standard deviations (SD), while categorical variables are presented as frequencies and percentages.

To evaluate changes in depressive and anxiety symptoms following rTMS treatment, paired-samples *t*-tests were performed comparing baseline and post-treatment scores on HAM-D and HAM-A scales.

Clinical outcomes were further assessed by calculating remission and response rates. Remission was defined as a post-treatment score of ≤7 on either the HAM-D or HAM-A. Response was defined as a reduction of ≥50% from baseline scores.

To explore variables associated with treatment response/remission, four multivariate logistic regression models were constructed, considering age, sex, educational level, family history of mental disorders, and baseline HAM-D and HAM-A scores as independent variables. Prior to running regression analyses, we assessed multicollinearity using variance inflation factors (VIF). Odds ratios (ORs) with 95% confidence intervals (CIs) and corresponding *p*-values were reported.

All statistical analyses were conducted using R (version 4.3.0; R Foundation for Statistical Computing, Vienna, Austria) [26]. The following R packages were used: tidyverse for data management and visualization [27], broom for model summarization [28], and ggpubr for graphical representations [29]. Statistical significance was set at *p* < 0.05.

## 3. Results

The final sample included 64 patients, predominantly female (75%), with a mean age of 54.22 years (SD = 15.38). Regarding educational attainment, 28.1% had completed primary education, 29.7% secondary education, and 28.1% held a university degree. Additionally, 61.0% of participants reported a positive family history of mental disorders. A full description of the sample’s sociodemographic and clinical characteristics is presented in Table 1.

**Table 1.** **Sociodemographic characteristics and clinical outcomes of the sample (N = 64)**.

Characteristic		N = 64
Age, mean (SD)		54.22 (15.38)
Female, n (%)		48 (75%)
Education, n (%)		
	Primary	18 (28.1%)
	Secondary	19 (29.7%)
	University	18 (28.1%)
Family history of mental disorders, n (%)		39 (61.0%)
Years since symptom onset, mean (SD)		21.58 (18.41)
HAM-D baseline, mean (SD)		25.86 (5.68)
HAM-D post-treatment, mean (SD)		13.66 (8.29)
HAM-A baseline, mean (SD)		30.25 (9.64)
HAM-A post-treatment, mean (SD)		16.45 (10.92)
Depression remission rate (HAM-D), n (%)		19 (30.0%)
Anxiety remission rate (HAM-A), n (%)		15 (23.0%)
Depression response rate (HAM-D), n (%)		29 (45.0%)
Anxiety response rate (HAM-A), n (%)		31 (48.0%)

HAM-D, Hamilton Depression Rating Scale; HAM-A, Hamilton Anxiety Rating Scale; SD, standard deviation.

**Fig. 2.**
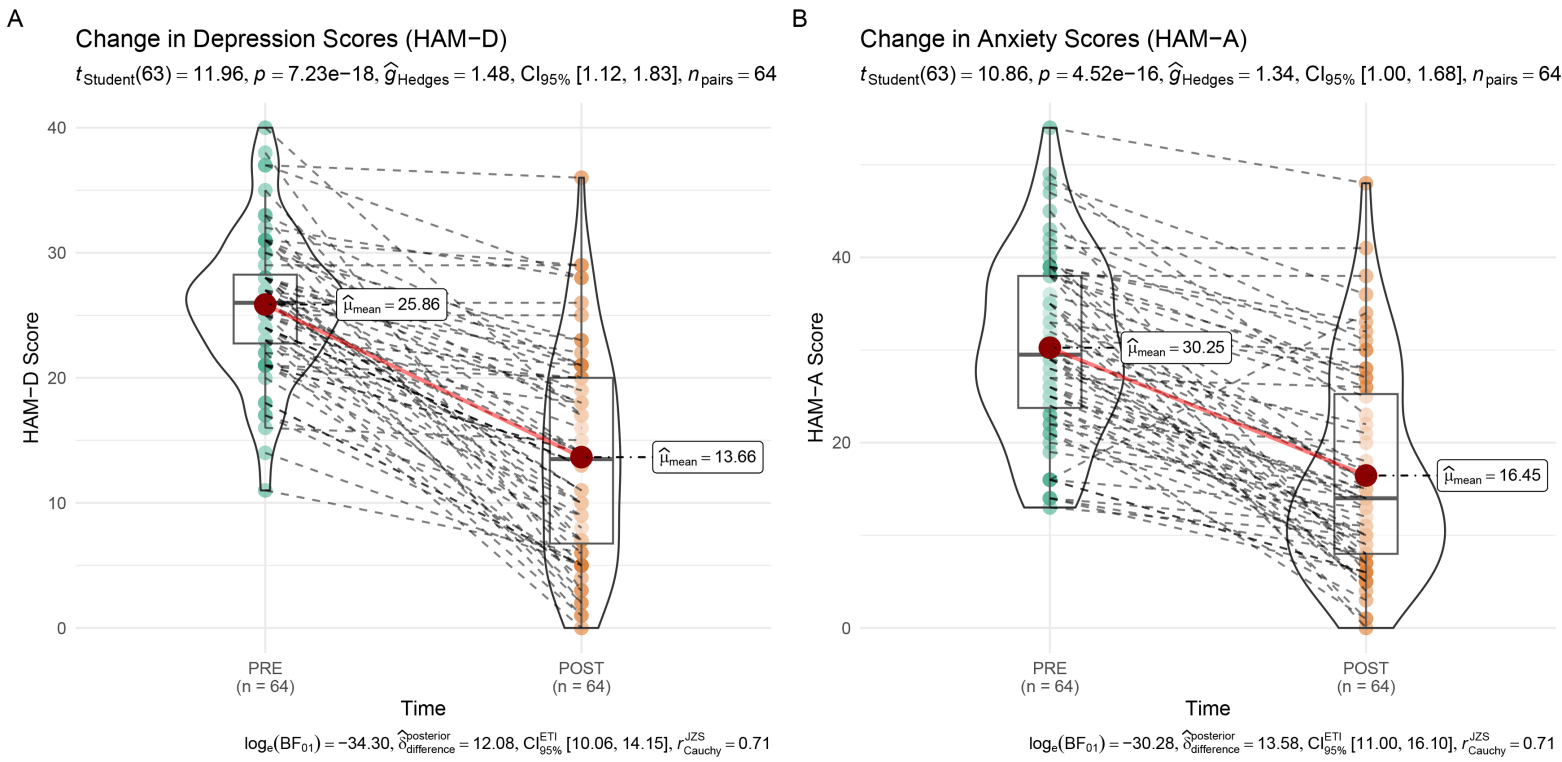
**Pre-post changes in depression and anxiety scores following TMS treatment**. (A) HAM-D and (B) HAM-A scores significantly decreased after the intervention. Violin plots represent score distributions; mean and confidence intervals highlight the treatment effect. Dashed lines connect individual patient scores. TMS, transcranial magnetic stimulation; HAM-D, hamilton depression rating scale; HAM-A, hamilton anxiety rating scale.

Following 30 sessions of TMS using the Seville Protocol, both depressive and anxiety symptoms showed significant reductions from pre- to post-treatment. As Shapiro-Wilk tests indicated non-normal distributions of change scores (HAM-D: W = 0.959, *p* = 0.031; HAM-A: W = 0.961, *p* = 0.041), non-parametric Wilcoxon signed-rank tests were applied. The results confirmed significant treatment effects, with reductions in HAM-D (V = 1891, *p* < 0.001, Hedges’ = 1.48, 95% CI [1.12, 1.83]) and HAM-A scores (V = 1909, *p* < 0.001, Hedges’ = 1.34, 95% CI [1.00, 1.68]). Full distributions and individual trajectories are displayed in Fig. 2A,B).

To test the robustness of the results, a sensitivity analysis was performed excluding participants with baseline HAM-A scores below 18 (n = 8). Wilcoxon signed-rank test confirmed statistically significant improvements in both depressive and anxiety symptoms (HAM-D: V = 1431, *p* < 0.001; HAM-A: V = 14.85, *p* < 0.001) from pre-to post-treatment.

In terms of clinical outcomes, response rates, defined as a ≥50% reduction from baseline scores, were 45.3% for depression (95% CI: 33.7–57.4) and 48.4% for anxiety (95% CI: 36.6–60.4). Remission rates, defined as a post-treatment score ≤7, was 29.7% for depression (95% CI: 19.9–41.8) and 23.4% for anxiety (95% CI: 14.7–35.1).

### 3.1 Tolerability and Adverse Events

The Seville Protocol was generally well tolerated. Given the retrospective nature of the study, only patients who completed the full acute course of treatment were included; therefore, no dropouts were recorded. Mild and transient side effects, including headache, scalp discomfort, and fatigue, were reported by 8 patients (12.5%). No serious adverse events were documented.

### 3.2 Variables Associated With Treatment Outcomes

To explore factors associated with treatment outcomes, multivariate logistic regression models were conducted using age, sex, educational level, family history of mental disorders, and baseline HAM-D and HAM-A scores as independent variables. All predictors included in the four logistic regression models exhibited VIF-adjusted values below 1.2, indicating low collinearity and acceptable independence of explanatory variables. Separate models were developed for response and remission outcomes, based on both depression (HAM-D) and anxiety (HAM-A) symptom change.

For depression response (HAM-D), the analysis revealed that patients with a positive family history of mental disorders were significantly associated with a lower likelihood of treatment response (OR = 0.226, 95% CI [0.05, 0.83], *p* = 0.033), corresponding to a 78% lower in the likelihood of responding to treatment compared to those without such a history. For depression remission, higher baseline HAM-D scores were marginally associated with a reduced likelihood of achieving remission (OR = 0.87, 95% CI [0.75, 1.01], *p* = 0.060), although this association did not reach statistical significance. However, neither association remained significant after FDR correction. No other variables in the model were significantly associated with response or remission (all *p* > 0.05).

In contrast, for anxiety outcomes, no significant predictors were identified for either response or remission.

A summary of the full results for all regression models is presented in Table 2. Forest plots illustrating the variables associated with both response and remission in depression and anxiety outcomes are provided in the **Supplementary Figs. 1,2,3,4**.

**Table 2.** **Logistic regression results for response and remission of depression and anxiety symptoms**.

Outcome	Predictor	β	Std. Error	95% CI (β)	*p*-value	FDR-adjusted *p*	OR (95% CI)
Depression response	(Intercept)	0.99	2.12	[−3.16, 5.28]	0.641	0.721	
	Age	−0.01	0.02	[−0.05, 0.04]	0.785	0.785	0.99 [0.95, 1.04]
	Sex (Male)	0.76	0.74	[−0.66, 2.28]	0.303	0.717	2.14 [0.52, 9.76]
	Education (Secondary)	0.60	0.77	[−0.89, 2.17]	0.433	0.717	1.82 [0.41, 8.77]
	Education (University)	−0.60	0.83	[−2.28, 1.01]	0.470	0.717	0.55 [0.10, 2.75]
	Family history (Yes)	−1.49	0.70	[−2.98, −0.18]	**0.033**	0.298	0.23 [0.05, 0.84]
	Years since onset	0.01	0.02	[−0.02, 0.05]	0.478	0.717	1.01 [0.98, 1.05]
	Baseline HAM-A	−0.04	0.03	[−0.11, 0.03]	0.299	0.717	0.96 [0.90, 1.03]
	Baseline HAM-D	0.04	0.06	[−0.08, 0.16]	0.562	0.721	1.04 [0.92, 1.17]
Anxiety response	(Intercept)	−2.08	2.03	[−6.22, 1.86]	0.307	0.691	
	Age	0.01	0.02	[−0.03, 0.06]	0.567	0.851	1.01 [0.97, 1.06]
	Sex (Male)	1.22	0.75	[−0.18, 2.79]	0.102	0.691	3.39 [0.84, 16.31]
	Education (Secondary)	0.83	0.73	[−0.59, 2.33]	0.258	0.691	2.29 [0.55, 10.26]
	Education (University)	0.01	0.80	[−1.60, 1.59]	0.992	0.992	1.01 [0.20, 4.91]
	Family history (Yes)	−0.67	0.64	[−1.98, 0.58]	0.299	0.691	0.51 [0.14, 1.79]
	Years since onset	−0.00	0.02	[−0.05, 0.03]	0.796	0.900	1.00 [0.95, 1.03]
	Baseline HAM-A	0.01	0.03	[−0.06, 0.07]	0.800	0.900	1.01 [0.94, 1.07]
	Baseline HAM-D	0.04	0.06	[−0.07, 0.16]	0.501	0.851	1.04 [0.93, 1.17]
Depression remission	(Intercept)	1.43	2.37	[−3.26, 6.21]	0.547	0.856	
	Age	0.02	0.03	[−0.03, 0.08]	0.353	0.836	1.02 [0.97, 1.08]
	Sex (Male)	−0.76	0.85	[−2.60, 0.83]	0.371	0.836	0.47 [0.07, 2.29]
	Education (Secondary)	0.19	0.78	[−1.34, 1.78]	0.810	0.912	1.21 [0.26, 5.94]
	Education (University)	−0.30	0.90	[−2.14, 1.48]	0.736	0.912	0.74 [0.12, 4.39]
	Family history (Yes)	−0.67	0.70	[−2.07, 0.72]	0.341	0.836	0.51 [0.13, 2.06]
	Years since onset	−0.00	0.02	[−0.04, 0.03]	0.913	0.913	1.00 [0.96, 1.03]
	Baseline HAM-A	0.02	0.04	[−0.05, 0.10]	0.571	0.856	1.02 [0.95, 1.10]
	Baseline HAM-D	−0.14	0.07	[−0.29, 0.01]	0.060	0.544	0.87 [0.75, 1.01]
Anxiety remission	(Intercept)	−2.37	2.44	[−7.51, 2.28]	0.333	0.804	
	Age	0.02	0.03	[−0.03, 0.08]	0.430	0.804	1.02 [0.97, 1.08]
	Sex (Male)	−0.41	0.85	[−2.23, 1.18]	0.625	0.804	0.66 [0.11, 3.25]
	Education (Secondary)	0.01	0.84	[−1.67, 1.71]	0.991	0.991	1.01 [0.19, 5.52]
	Education (University)	0.70	0.90	[−1.04, 2.55]	0.433	0.804	2.01 [0.35, 12.87]
	Family history (Yes)	−0.60	0.71	[−2.00, 0.83]	0.400	0.804	0.55 [0.14, 2.30]
	Years since onset	0.00	0.02	[−0.04, 0.04]	0.913	0.991	1.00 [0.96, 1.04]
	Baseline HAM-A	−0.03	0.04	[−0.10, 0.04]	0.448	0.804	0.97 [0.90, 1.04]
	Baseline HAM-D	0.04	0.06	[−0.08, 0.17]	0.540	0.804	1.04 [0.92, 1.19]

Note. β = logistic regression coefficient; OR, odds ratio; CIs, confidence intervals. OR and their 95% CIs were computed as the exponential of the β coefficients and their 95% confidence intervals. *p*-values were adjusted using the Benjamini-Hochberg false discovery rate (FDR) correction. Statistically significant associations (*p* < 0.05) are shown in bold.

## 4. Discussion

This study evaluated the effectiveness of an accelerated, high-intensity bilateral TBS protocol (the Seville Protocol) for TRD with moderate-to-severe comorbid anxiety symptoms. Our results demonstrated substantial reductions in depressive and anxiety symptoms after 30 sessions of bilateral TBS, with large effect sizes for both outcomes, depressive and anxiety symptoms. Remission and response rates were also clinically relevant.

The Seville Protocol, with its intensified stimulation parameters (higher intensity, increased pulses per session, and accelerated delivery), appears to be a feasible and effective alternative to standard rTMS protocols. In contrast to FDA-approved approaches such as 10 Hz rTMS or unilateral iTBS, the Seville Protocol employs bilateral stimulation with higher intensities (133.5% RMT for iTBS), guided by electric field modeling to enhance prefrontal engagement [15]. Notably, the Seville Protocol achieved a remission rate of 30%, which is comparable to the 37% reported in standard FDA-approved protocols [23, 30], despite its significantly shorter duration. Whereas conventional rTMS protocols typically span 4–6 weeks of daily sessions, the Seville Protocol is completed in just 3 weeks, with three to four sessions per day across only nine treatment days. This condensed schedule not only reduces the overall duration of treatment but also likely decreases indirect costs associated with prolonged care, such as missed workdays, transportation, and caregiver time, making it more accessible and economically viable for healthcare systems and patients alike. The accelerated design may also enhance adherence and patient satisfaction, particularly in populations with limited availability or logistical constraints. Nonetheless, it is important to note that even shorter protocols have been explored. For instance, the ONE-D protocol developed by Nanos [31] delivered 20 iTBS sessions in a single day, achieving a 62.5% response and 37.5% remission rate in a real-world primary care setting. While promising, this approach also included the off-label use of d-cycloserine and lisdexamfetamine to enhance neuroplasticity and was tested in a small retrospective sample.

Another high efficacy accelerated protocol for patients with TRD is the SAINT protocol [19], which showed remission rates of up to 79% in depressive symptoms among TRD populations [19] applying unilateral iTBS to the left DLPFC, using individualized targeting based on functional connectivity MRI (fcMRI) to identify the region most anti-correlated with the subgenual anterior cingulate cortex (sgACC). The protocol consists of 10 sessions per day for 5 consecutive days, with each session delivering 1800 pulses (totaling 90,000 pulses), at 90% of the RMT and capped at a maximum of 120% [18]. While SAINT achieves remarkable clinical outcomes, its implementation may be limited in certain contexts due to logistical and economic demands, such as the need for fcMRI-based targeting, continuous high-frequency scheduling, and specialized infrastructure. In contrast, the Seville Protocol proposes a more scalable alternative for patients with TRD and comorbid anxiety, delivering similarly intensive stimulation across a condensed three-week, guided by standard neuronavigation systems and without the need for functional imaging, thus reducing overall cost and complexity. Notably, recent findings by Morriss *et al*. [32] suggests that fcMRI targeting may not offer clear clinical advantages over standard structural neuronavigation. While more precise, its added complexity and cost may not be justified.

In terms of anxiety, FDA-approved rTMS protocols such as high-frequency left DLPFC or low-frequency right DLPFC stimulation have shown mixed results, with only limited efficacy in treating comorbid anxiety symptoms, especially when compared with the H1 Coil, which demonstrated significantly greater effect sizes [33]. Notably, the Seville Protocol, despite employing a double-cone coil, which is typically associated with more focal and superficial stimulation, yielded a 48.4% response and 23.4% remission rate for comorbid anxiety symptoms, outcomes that exceed those reported in most standard TMS interventions. For example, the bilateral accelerated TBS protocol tested by Chen *et al*. [16], reported a response rate of 44.1 and 36.8% and a remission rate of 26.9 and 24.1% for depression after, bilateral TBS at 80 % or 120 % RMT respectively, across 20 sessions over four weeks; although anxiety was also assessed, categorical response or remission rates for these symptoms were not provided. Similarly, Clarke *et al*. [34], in a large naturalistic study of 248 patients with TRD, including 172 with comorbid anxiety disorders, found that bilateral rTMS (10 Hz left and 1 Hz right at 110% RMT) produced a 39.5% response and 23.3% remission rate for anxiety symptoms, with remission rates comparable but response rates lower than those observed in the present study. Thus, the higher pulse dose, increased stimulation intensity, and accelerated schedule employed in the Seville Protocol may contribute to its superior clinical outcomes, along with enhanced tolerability and applicability in real-world clinical contexts.

While some participants (n = 8) had HAM-A scores slightly below the commonly accepted threshold for moderate anxiety, we included them to reflect the clinical heterogeneity of real-world TRD populations. Eligibility was based on the psychiatrist’s judgment of moderate-to-severe anxiety with functional impairment, rather than a strict numerical cut-off. This approach aligns with the naturalistic design of the study and enhances external validity. To account for potential bias, a sensitivity analysis excluding these cases was performed and confirmed significant improvements in both depression and anxiety, reinforcing the robustness of our findings. Future trials may benefit from standardized thresholds while balancing ecological validity.

Recent neuroimaging studies have shown that an enlarged frontostriatal salience network, involving the anterior cingulate cortex, insula, and striatum, is a stable feature in individuals predisposed to depression, detectable even before clinical onset, and is linked to development of anhedonia and anxiety [35]. The Seville Protocol combines iTBS to the left and cTBS to the right DLPFC, aiming to increase cortical excitability in the left prefrontal regions while decreasing excitability in the right. This bilateral approach is theoretically grounded in the interhemispheric imbalance model of affective disorders, which postulates relative hypoactivity of the left DLPFC and hyperactivity of the right DLPFC, withing the broader context of generalized prefrontal hypofunction observed in depression [36, 37]. By modulating excitability in both hemispheres and affecting in network- connectivity level, the Seville Protocol may help restore functional balance within prefrontal-limbic networks implicated in emotion regulation.

Regarding baseline sociodemographic and clinical, the logistic regression analysis suggested that a positive family history of mental disorders was associated with a lower probability of depression response. However, this effect did not remain significant after FDR correction. None other baseline variables, including age, sex, education level, or baseline severity, were associated with treatment outcomes for either depression or anxiety. These results are consistent with recent efforts to characterize predictors of rTMS efficacy. For example, Benster *et al*. (2025) [38] applied machine-learning techniques to electronic medical records and found that comorbid anxiety, obesity, benzodiazepine or antipsychotic use, and longer episode duration were linked to poorer outcomes, whereas trauma history, former tobacco use, iTBS protocols, and a higher number of sessions were associated with improved response. Similarly, Trevizol *et al*. (2020) [11], using data from the large THREE-D trial, reported that higher baseline severity and greater refractoriness were associated with lower remission rates, while current employment increased the likelihood of remission. In line with our exploratory signal, Calvet *et al*. (2017) [39] also identified psychiatric family history as a negative predictor of rTMS response in a naturalistic cohort. Taken together, these findings highlight the complexity of prognosticating rTMS outcomes and suggest that single clinical variables are unlikely to provide robust prediction. Future research should therefore prioritize large-scale, prospective studies that integrate clinical and demographic data with biological markers and advanced predictive modeling to better personalize neuromodulation treatments.

This study presents several strengths and limitations. Among the strengths, it is noteworthy that the sample consisted of patients with TRD and comorbid anxiety symptoms, a combination highly prevalent in clinical practice but often excluded from trials. However, several limitations must be acknowledged. First, the retrospective design limits causal inference and increases the risk of selection and reporting bias. Second, the absence of a sham-controlled or comparison group prevents definitive conclusions regarding the specificity of treatment effects and may overestimate clinical impact, even though large within-subject improvements were observed. Third, only patients who completed the full treatment protocol were included, and no dropouts were reported, which may have introduced selection bias favouring those who tolerated and responded to treatment. Additionally, the modest sample size and demographic skew toward middle-aged females further restrict external validity. Lastly, the lack of predefined HAM-D cut-off scores and the use of clinician judgment for anxiety inclusion criteria, while aligned with real-world practice, may reduce comparability across studies. In addition, although widely used, both the HAM-D and HAM-A scales have recognized limitations, including symptom overlap and some degree of inter-rater variability. While these limitations should be considered when interpreting the results, the findings may inform future hypothesis-driven research in larger and controlled trials including long-term follow-up.

## 5. Conclusions

The Seville Protocol, which combines accelerated, high-intensity bilateral TBS, was associated with significant and clinically meaningful reductions in both depressive and anxiety symptoms in patients with TRD. These findings suggest that protocol may offer a viable therapeutic option for patients with comorbid moderate-to-severe anxiety, especially in settings seeking shorter treatment durations. Nonetheless, to establish its efficacy and broader applicability, future research should prioritize larger, prospective randomized controlled trials, including comparisons with both sham and standard TBS protocols, as well as studies into potential biomarkers of treatment response.

## Availability of Data and Materials

The datasets generated and analyzed during the current study are available in the https://docs.google.com/spreadsheets/d/1ujB3aCo4mJloevNRpHY8OBOw20uIqR2f/edit?usp=drive_link&ouid=109819323182051588938&rtpof=true&sd=true.

## Supplementary Material

Supplementary material associated with this article can be found, in the online version, at https://doi.org/10.31083/AP45254.
