# Supplementary material for: Enhanced Bilateral Accelerated Theta Burst Stimulation for Comorbid Anxiety and Depressive Disorders: The Seville Protocol
Source: Alpha Psychiatry. 2025 Dec 16;26(6):45254. doi: 10.31083/AP45254 (PMC12781213; doi:10.31083/AP45254)
Supplement: Supplementary file 1 [file 2757-8038-26-6-45254-s1.docx]

**Supplementary Materials**

**Supplementary Fig. 1**. Forest plot displaying odds ratios (ORs) and 95% confidence intervals (CIs) from a multivariate logistic regression predicting response based on depression scores (HAM-D). Predictors include age, sex, educational level, family history of psychiatric disorders, baseline HAM-D, and baseline HAM-A. The dashed vertical line represents the null effect (OR = 1).


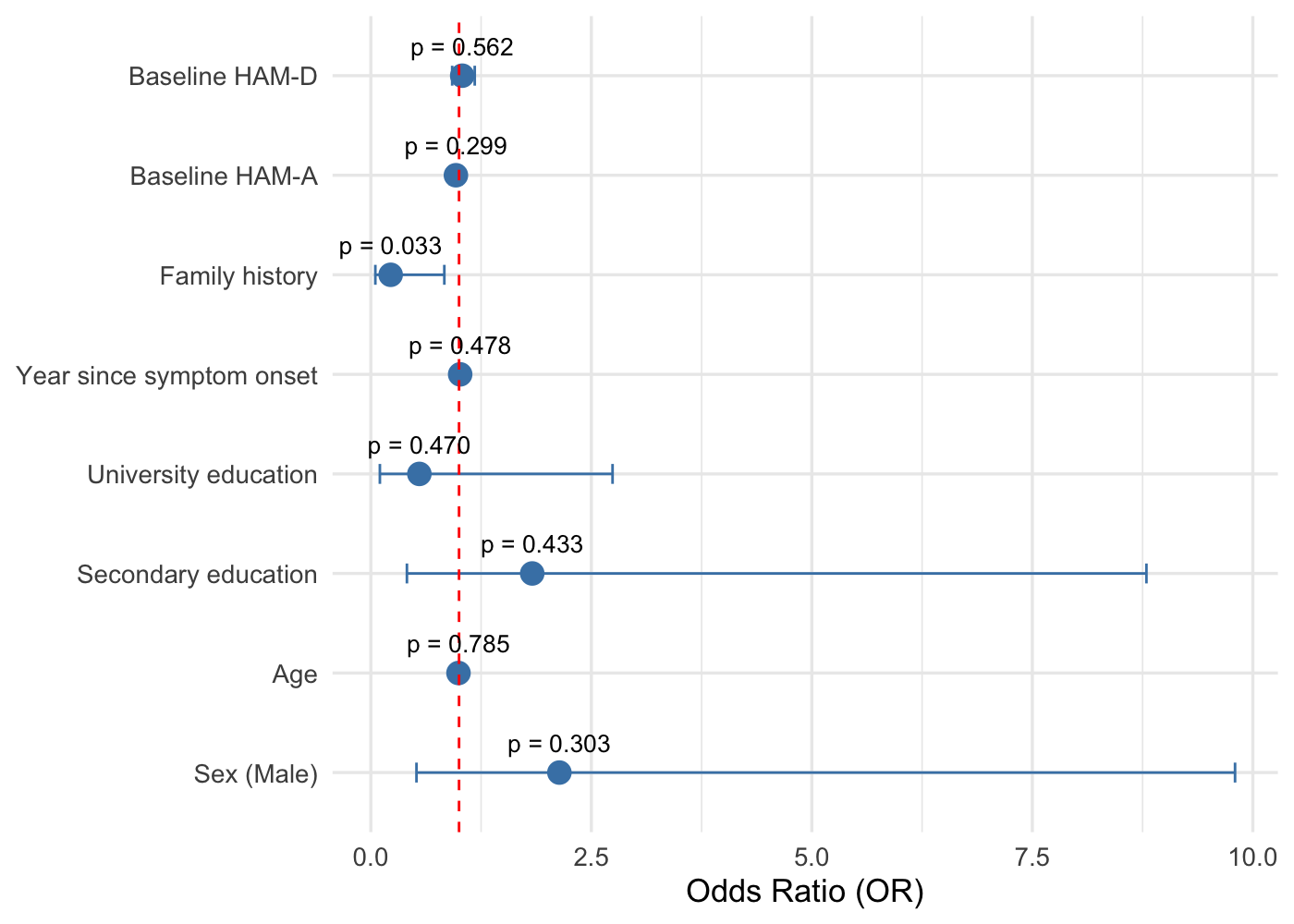


**Supplementary Fig. 2**. Forest plot summarizing ORs and 95% CIs for predictors of anxiety response (HAM-A). No significant associations were observed. Variables included match those in previous models. The dashed vertical line represents the null effect (OR = 1).


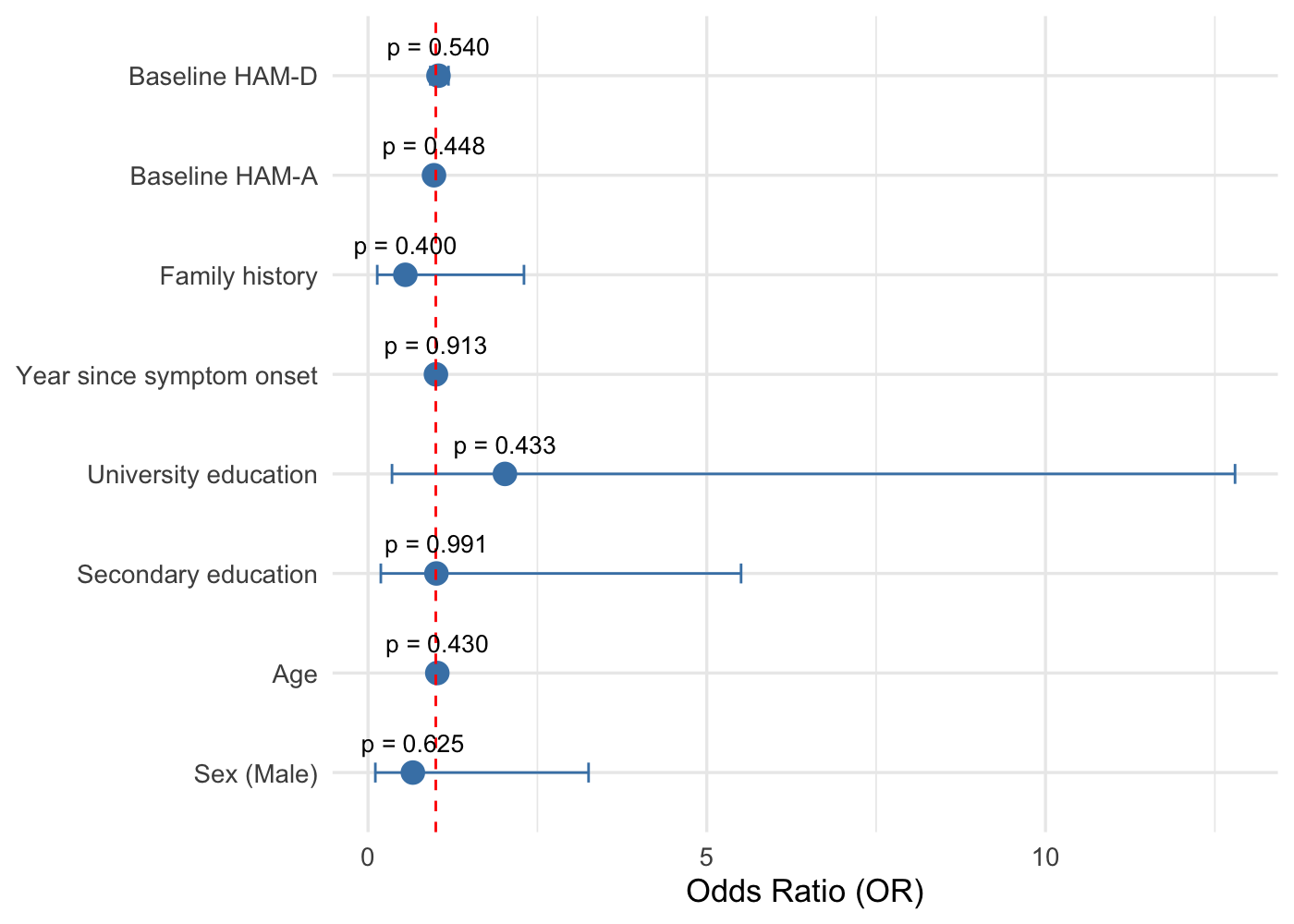


**Supplementary Fig. 3**. Multivariate regression model for depression remission (HAM-D), with forest plot showing ORs and 95% CIs. A trend-level association was observed for baseline HAM-D (p = 0.060); however, this did not remain significant after correction for multiple comparisons (FDR). The dashed vertical line represents the null effect (OR = 1).


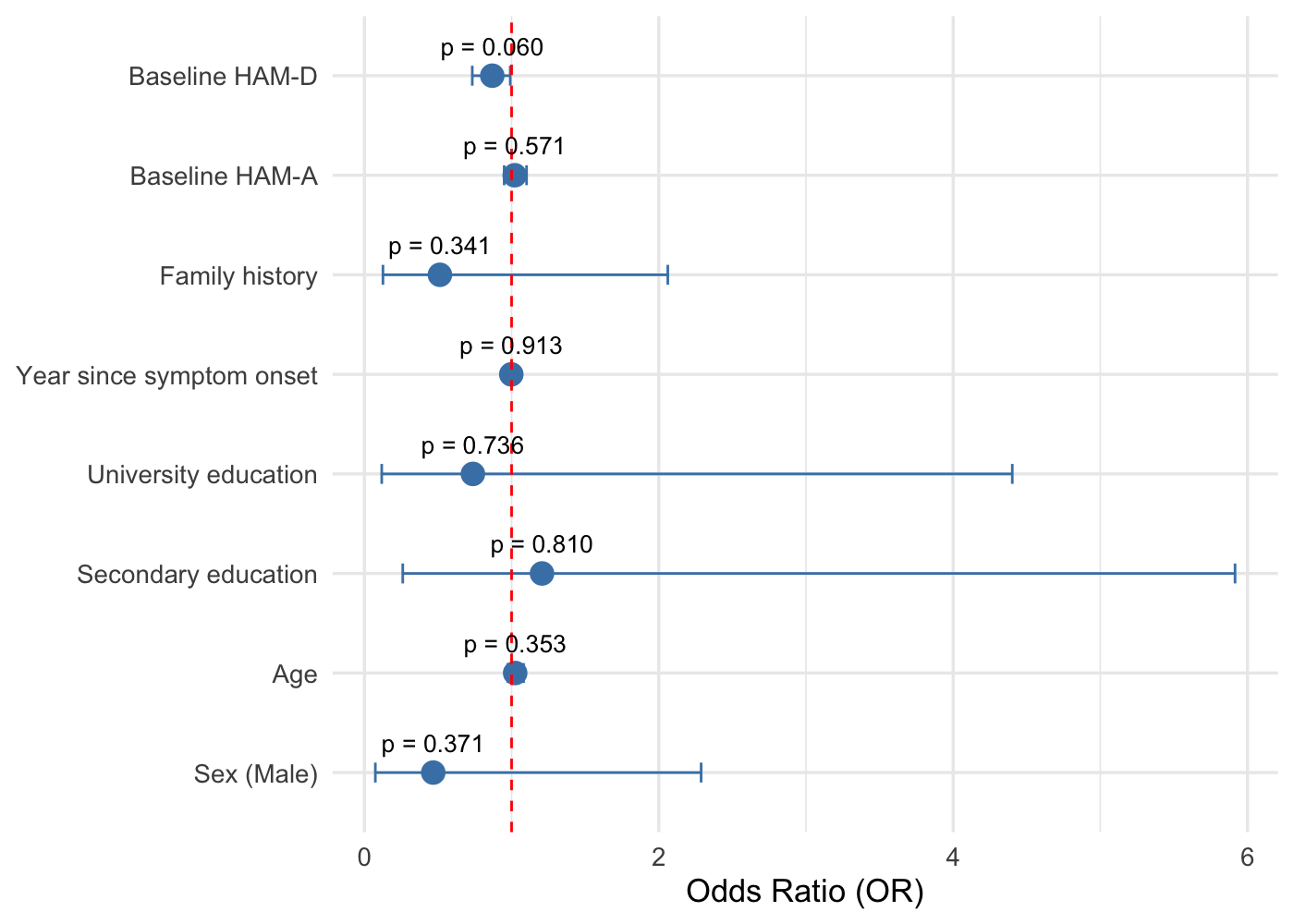


**Supplementary Fig. 4.** Forest plot of ORs and 95% CIs for predictors of anxiety remission (HAM-A). None of the predictors were statistically significant (*p* > 0.05). Covariates included are the same as in Supplementary Figure 1. The dashed vertical line represents the null effect (OR = 1).

*
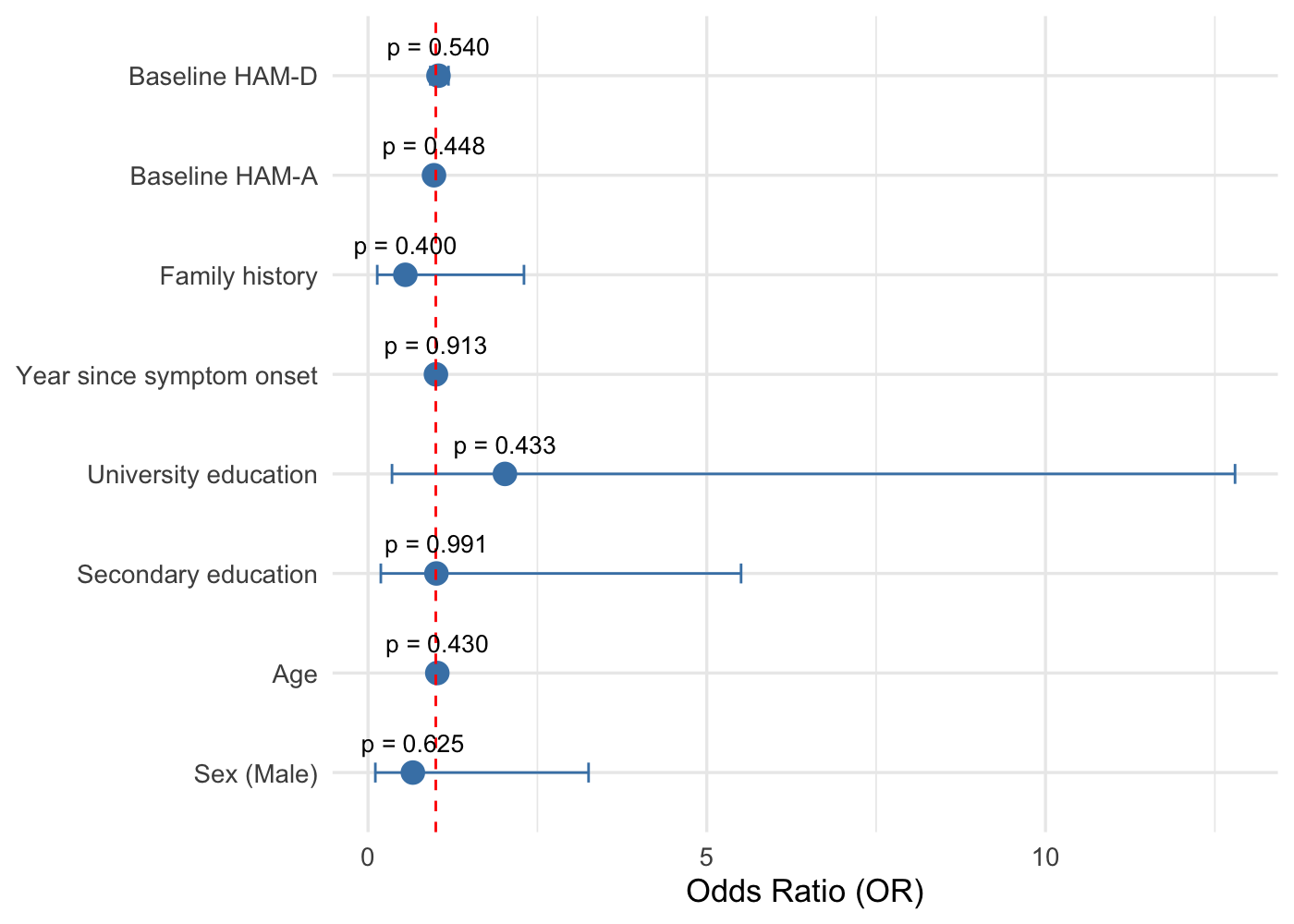
*
